# Hypoxia-Inducible Factor-1α: The Master Regulator of Endothelial Cell Senescence in Vascular Aging

**DOI:** 10.3390/cells9010195

**Published:** 2020-01-13

**Authors:** Matilde Alique, Elsa Sánchez-López, Guillermo Bodega, Chiara Giannarelli, Julia Carracedo, Rafael Ramírez

**Affiliations:** 1 Departamento Biología de Sistemas, Facultad de Medicina y Ciencias de la Salud (IRYCIS), Universidad de Alcalá, Alcalá de Henares, 28805 Madrid, Spain; manuel.ramirez@uah.es; 2 Departments of Pharmacology and Pathology, University of California San Diego, La Jolla, CA 92037, USA; esl023@health.ucsd.edu; 3 Departamento de Biomedicina y Biotecnología, Facultad de Biología, Química y Ciencias Ambientales, Universidad de Alcalá, Alcalá de Henares, 28805 Madrid, Spain; guillermo.bodega@uah.es; 4 Cardiovascular Research Center, Institute for Genomics and Multiscale Biology, New York, NY 10029, USA; chiara.giannarelli@mssm.edu; 5 Precision Immunology Institute, Icahn School of Medicine at Mount Sinai, One Gustave L. Levy Place, New York, NY 10029, USA; 6 Departamento de Genética, Fisiología y Microbiología, Facultad de Biología, Universidad Complutense de Madrid, 28040 Madrid, Spain; 7 Instituto de Investigación Sanitaria Hospital 12 de Octubre (i+12), 28041 Madrid, Spain

**Keywords:** hypoxia-inducible factor-1α (HIF1α), vascular aging, senescent cells, endothelial cells, vascular smooth muscle cells, atherosclerosis, extracellular vesicles

## Abstract

Aging is one of the hottest topics in biomedical research. Advances in research and medicine have helped to preserve human health, leading to an extension of life expectancy. However, the extension of life is an irreversible process that is accompanied by the development of aging-related conditions such as weakness, slower metabolism, and stiffness of vessels. It also debated that aging can be considered an actual disease with aging-derived comorbidities, including cancer or cardiovascular disease. Currently, cardiovascular disorders, including atherosclerosis, are considered as premature aging and represent the first causes of death in developed countries, accounting for 31% of annual deaths globally. Emerging evidence has identified hypoxia-inducible factor-1α as a critical transcription factor with an essential role in aging-related pathology, in particular, regulating cellular senescence associated with cardiovascular aging. In this review, we will focus on the regulation of senescence mediated by hypoxia-inducible factor-1α in age-related pathologies, with particular emphasis on the crosstalk between endothelial and vascular cells in age-associated atherosclerotic lesions. More specifically, we will focus on the characteristics and mechanisms by which cells within the vascular wall, including endothelial and vascular cells, achieve a senescent phenotype.

## 1. Aging, Vascular Senescence, and Cardiovascular Diseases

Aging is an irreversible process that arises due to the accumulation of harmful or deleterious changes that occur in cells and tissues over time and affects the physiological functions necessary for survival and homeostasis [1,2]. Colloquially, aging is defined as the process of becoming older. Presently, in the 21st century, there is still a debate as to whether we should consider aging a disease *per se*, or a natural, ineluctable “healthy” process. It is becoming more evident that aging, as a decline in the optimal functions of cells and organs, may in fact be considered a disease *per se*, which establishes a preconditioned state for some aging-associated diseases, including cancer, retinal pathologies, osteoporosis, osteoarthritis, chronic kidney disease (CKD), hypertension (HTA), diabetes *mellitus* type 2 (DMII), and cardiovascular diseases (CVD), especially atherosclerosis [3]. Indeed, the development of atherosclerosis is defined as a premature aging disorder and is the leading cause of death among people over 65 years of age. One aspect that actively contributes to the development of atherosclerosis and other CVD, is vascular aging, which has become an active field of research in the last decade [4]. In vascular aging, the arterial vascular wall is affected by an increase in both senescent endothelial cells (ECs) as well as vascular smooth muscle cells (VSMCs) [5]. The exact mechanism that mediates ECs to become senescent in the vascular wall is still not fully understood; however, much evidence has revealed a crucial role for hypoxia-inducible factor-1α (HIF-1α) in the development of the senescent phenotype and the progression of atherosclerosis [6,7,8,9,10].

Cellular aging or cellular senescence is a process that occurs naturally in our cells when we age, due to persistent exposure to cellular stressors [11]. Senescent cells are characterized by a permanent state of cell cycle arrest accompanied by a secretory phenotype and resistance to cell death [11]. When a senescent phenotype becomes dysfunctional, the ability to maintain homeostasis between living and dead cells is lost. Senescent cells appear naturally during aging, also known as healthy aging, and during aging linked to age-related diseases as a result of pathological stress. While senescent cells appear during embryogenesis and in healthy young organs during tissue remodeling and repair, the accumulation of senescent cells in tissues, including the vasculature, is a hallmark of aging [12].

Moreover, these cells lose their proliferative capacity and acquire a new phenotype. In fact, senescent cells (that also cease to proliferate and undergo other functional changes in association with aging) are characterized by oxidant and inflammatory phenotypes. The senescent phenotype confers cells a flattened and enlarged appearance, with a persistent non-dividing state that is metabolically active and characterized by significant reorganization of protein expression and secretory programs, which ultimately result in the senescence-associated secretory phenotype (SASP) [13]. SASP occurs regardless of the stimulus that initiates senescence, such as a replicative senescence model in vitro, aging in vivo, and even when senescent cells appear as a consequence of stressful stimuli, known as stress-induced premature senescence (SIPS) [14]. SIPS is considered a reversible phenomenon [7]. In any case, the secretory phenotype includes the secretion of cytokines, growth factors, proteases, and extracellular vesicles (EVs), which exert a paracrine function affecting neighboring cells and propagate the senescent phenotype [15]. Notably, depending on the context, many of these secreted factors also support chronic inflammation and cellular transformation.

In mammals, aging is associated with the accumulation of senescent cells [8]. In the case of age-related chronic diseases, senescent cells are also accumulated, and some studies have demonstrated that the accumulation of senescent cells has a causative role in the pathology of these age-related disorders [16]. Senescent cells are characterized by the loss of their physiological resilience. In other words, through aging and pathogenic signals in chronic diseases and conditions, cells are exposed to a new microenvironment and become senescent due to their inability to adapt [17]. Cellular senescence clearly drives various pathological changes associated with aging [8]. Indeed, delaying senescent cell accumulation could have a protective role in the development of chronic diseases. This new concept, based on the studies of molecular mechanisms in the evolution of cellular senescence to avoid age-associated diseases, offers a new approach to secure the survival of senescent cells [1]. For this purpose, senotherapy (or senolysis) has been coined for therapies where senolytic drugs, which are intended to eliminate senescent cells by apoptosis [1,7,8,17,18], have shown beneficial effects in experimental models and human clinical trials [19,20,21,22,23]. Senolytic drugs may be useful as inhibitors of the progression of aging *per se* or disorders associated with premature aging, such as CVD [18,24,25].

Vascular aging causes several associated diseases as a consequence of advanced age; in fact, aging could be considered a critical risk factor for vascular damage. For instance, arterial stiffness is associated with changes in blood pressure [26]. Endothelial dysfunction induces high peripheral vascular resistance until about age 50 [27]. This phenomenon causes high systolic blood pressure that increases arterial stiffness, thereby initiating a vicious cycle. Moreover, in older normotensive people, aging produces higher arterial stiffness, systolic blood pressure, and pulse pressure [26,27]. These observations may be related to the increased accumulation of senescent cells in the vascular wall in aging or age-related CVD [18]. Indeed, vascular cellular senescence contributes to the pathogenesis of human arterial aging [28], and endothelial cell senescence has been reported to develop in heart failure and promote pathologic changes in a failing heart [8].

The current characterization of senescent cells includes the evaluation of molecular senescence markers, such as p53, p21, p16, and γ-H2AX, as well as telomere attrition, which are enhanced signals for senescence-associated beta-galactosidase (SA-β-gal). These markers are indirect indicators because the activation of these pathways is simply one piece of evidence that cells are senescent [11]. Interestingly, once the p16 pathway is activated, senescence arrest cannot be reversed by the inactivation of p53 or the silencing of p16 [16]. A senescent cell uses a different process to achieve a senescent phenotype; therefore, this type of cell must be defined through various combinations of markers [18]. The structural and biological changes in the vascular walls that are linked to a specific mechanism in age-related changes of the vasculature are not yet well defined, mainly because they lack specific biomarkers for aging, which makes the study of vascular aging challenging. Along these lines, emerging evidence has described a new mechanism in senescence progression, where HIF-1α and the microRNA (miRNA) miR-126 play a critical role in senescent ECs, proposing HIF-1α and miR-126 as new markers for endothelial senescence progression [29]. Moreover, miR-126 are implied in other physio-pathological processes: (1) both miR-126 strands are involved as an anti-inflammatory target in controlling leukocyte interaction with human brain endothelium under shear stress conditions [30]; (2) neuronally expressed miR-126-5p regulates angiogenesis by protecting endothelial cells of the developing retinal vasculature from apoptosis [31]; (3) miR-126 is essential for endothelial phenotype expression, promoting endothelial differentiation in autograft adipose-derived stem cells [32] and highlighting that miR-126 from endothelial cells and exosomes from these cells present neurorestorative effects in DMII mice [33]; and, finally, miR-126 presents a protective effect during hypoxia/reoxygenation in endothelial cells mediated by the PI3K/Akt/endothelial nitric oxide synthase (eNOS) signaling pathway [34].

## 2. Hypoxia-Inducible Factors

Hypoxia-inducible factors (HIFs) are the product of a heterodimeric transcriptional factor subdivided into three-member families of nuclear transcriptional factors (HIF1, HIF2, and HIF3), each one including α and β (nuclear translocator) subunits. HIF is a constitutive factor that is evolutionary conserved and plays a critical role in oxygen (O_2_) homeostasis from invertebrates up to humans.

Moreover, HIF is involved in the physiological mechanisms that lead to homeostasis, such as energy metabolism, cell growth, survival, invasion, migration, and angiogenesis [35]. O_2_ levels regulate both HIF-1α and HIF-1β (or aryl hydrocarbon receptor nuclear translocator; ARNT) and associate with DNA-binding transcription factor proteins. HIF-1α is a ubiquitous factor expressed in nucleated cells, while HIF-2α and HIF-3α are cell type-specific factors that are mainly expressed in vascular endothelial cells (ECs), renal cells, liver cells, and some cells from the myeloid lineage [36,37].

HIF-1α performs its critical function in the maintenance of vascular homeostasis [38] by orchestrating angiogenesis and vascular wall repair [29,39,40,41]. In addition to its role in the formation of blood vessels for O_2_ delivery, HIF also regulates erythropoietin (EPO) production in the kidneys that, in turn, controls red blood cell production. Further, more than one hundred genes have been identified to be governed by mammalian HIF during O_2_ deprivation [42,43]. The involvement of HIF-1α in diseases of the vascular wall, including atherosclerosis, carotid stenosis, and aneurysms, has recently been proposed [44,45,46,47,48,49]. Indeed, insufficient expression of HIF is associated with CVD [50,51]. Moreover, short-term HIF overexpression has beneficial effects on the heart, and long-term HIF stabilization is associated with cardiomyopathy [52]. Recent emerging data reveal crosstalk between HIF-1α and cellular senescence-associated cardiovascular aging (Table 1) [3,29,52,53,54].

## 3. The Physio-Pathological Role of HIF in Aging

Since the discovery of HIF-1α, several seminal works have identified the changes in HIF associated with age and the development of age-related disorders [55], including neurodegenerative diseases [56]. Importantly, in 2009, Mehta et al. described HIF-1 as a longevity factor, demonstrating that HIF-1 stabilization is associated with a 30–50% increase in lifespan [57]. Several studies have shown that stabilization of HIF-1 increases longevity and healthspan through different pathways in *Caenorhabditis elegans* [58,59]. These critical findings in worms yielded a new perspective on the study of HIF stabilization and lifespan among mammals [60]. However, the stabilization of mammalian HIF-1α has been implicated in tumor growth and cancer development and may therefore be harmful. Consequently, a balance between the beneficial and detrimental effects of HIF is critical for homeostasis and depends on the involved components and their contribution to longevity. Members of the p53 family counteract HIF stability; as a consequence, the O_2_-independent regulation of HIF-1 impacts the tumorigenic potential of cancer cells, thereby affecting angiogenesis, metabolism, and metastasis [61].

Studies on skin, a tissue that is continuously exposed to intrinsic and extrinsic aging factors, have identified HIF-1α as a crucial determinant of skin homeostasis, especially in epidermal aging and wound healing [62]. Results have reported that the loss of epidermal HIF-1α accelerates epidermal aging and affects re-epithelialization in humans and mice [62]. Notably, significant elevations in both hypoxia-inducible transcription factors HIF-1α and HIF-1β gene expression have also been found in the gingival tissues of aged animals, even though these tissues were deemed clinically healthy [63]. In a model of limb ischemia in mice, HIF-1 was found to mediate angiogenesis and, therefore, has been proposed to contribute to the pathological aging process [54].

Aging *per se* is a consequence of other diseases called age-related pathologies, because the risk of organ failure and age-associated disease increases with the advance of age [1]. Atherosclerosis is primarily featured by senescent cells in advanced human atherosclerotic plaques, and aging is considered to be a dominant risk factor for disease development [64]. Indeed, some studies have shown that markers of senescent cells are found in atherosclerotic plaques but not in non-atherogenic arteries [5,65]. Moreover, the arteries from older healthy adults and hypertensive patients have higher expression of p21, a marker of senescence, as well as the SASP pro-inflammatory phenotype [66]. In the vascular wall, the SASP phenotype is responsible for the enrichment of the pro-inflammatory milieu. Both interleukin (IL)-6 and IL-8, through a paracrine effect, cause negative feedback over cellular growth and contribute to maintenance of the senescent phenotype [67]. Notably, in this case, the molecular program of senescence is HIF-dependent [68]. Likewise, ECs sampled from healthy older adults have higher expression of the senescence markers p21 and p16, both of which are correlated with blunted endothelial function [69].

By contrast, older adults that perform regular exercise, which may be considered a vasoprotective lifestyle factor, have normalized endothelial senescent markers and endothelial function [5]. Moreover, some mediators, such as miRNAs, are described to have a critical role in (patho)physiological processes, including atherosclerosis [70,71,72]. In particular, miR-126 exerts an atheroprotective effect [72,73] due to its role in restoring endothelium homeostasis [74].

Concretely, miR-126 modulates stromal cell-derived factor-1 (SDF-1) and vascular cell adhesion molecule-1 (VCAM-1) in ECs [75]. During endothelial dysfunction, miR-126 levels are decreased and as a consequence, an augmented SDF-1 expression improves stroke outcome [75,76]. However, miR-126 modulates C-X-C chemokine receptor type 4 (CXCR4) protein levels during atherosclerosis. As a result, SDF-1 is released and increase the recruitment of endothelial progenitor cells to the atheroma plaque [77]. Nowadays, miR-126 has been described as a dual regulator of atherosclerosis [77]. On the one hand, miR-126 induced SDF-1, which promoted endothelial progenitor cells during atheroma formation. As a consequence, VCAM-1 expression is inhibited and EC proliferation limited atherosclerosis progression. In this way, atherosclerosis formation is impeded and miR-126 shows a beneficial role in atherosclerosis [76,77]. On the other hand, few studies showed that miR-126 presents adverse effects in atherosclerosis. Mir-126 affects the proliferation and apoptosis of VSMCs and also these cells do not express miR-126 during atherosclerosis [78]. In contrast, Hao and Fan showed that miR-126 is upregulated in atherosclerosis lesions in mice [79]. In conclusion, the presence of miR-126 in ECs has beneficial effects in atherosclerosis, whereas miR-126 in VSMCs exacerbated atherosclerosis.

Despite the complex relationship between HIF and aging, Kaluz et al. recently noted that several age-associated maladies involve the disarray or activation of the hypoxia response mechanism, mainly the HIF transcriptional factor [3]. In part, this phenomenon could be related to evidence for the overactivation of hypoxia responses to a modestly slow aging life, associated with beneficial stress responses in various animal studies. These findings support the idea of targeting HIF in early age-related diseases to slow down aging and prevent the progression of aging-related diseases. The authors concluded that an “HIF pill” could be preventive in the development of early age-related disease, thereby slowing the progression of aging. Recently, Semenza [80] summarized the HIF stabilizers registered in clinical trials for anemia, a common complication of CKD. We propose that HIF stabilization with the treatment of these drugs could help prevent age-related disease.

**Figure 1:**
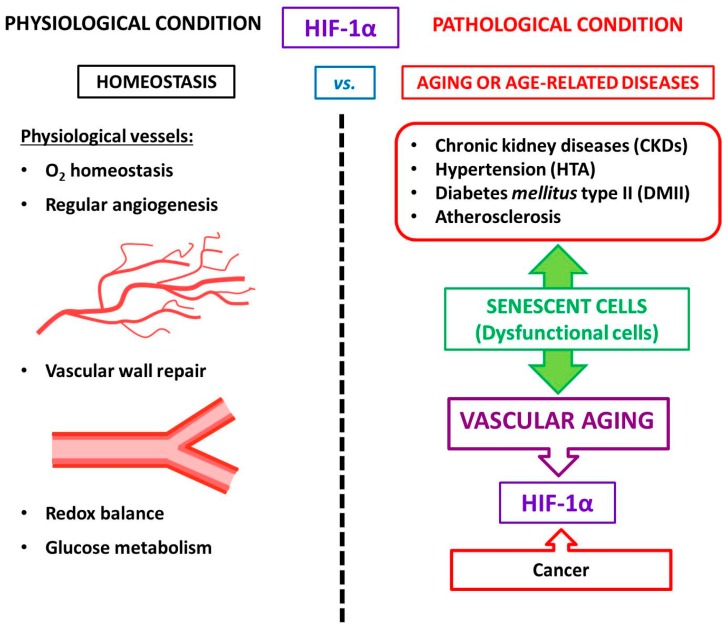
Schematic illustration of HIF’s role in homeostasis (physiological conditions) compared with the pathological situation (age-associated diseases included).

We summarize the role of HIF under physiological and pathological conditions (age-associated diseases included) in Figure 1.

## 4. HIF-1α and Vascular Aging

As mentioned above, HIF is not only a transcriptional factor that regulates tissue oxygenation (including angiogenesis and vascular remodeling) but also controls redox balance, inflammation, and glucose metabolism to eventually maintain cellular homeostasis [36,61].

According to current knowledge, the age-dependent impairment of HIF-1α induction leads to diminished vascular responses to limb ischemia [54] and less effective wound healing [81]. Some evidence shows the functionally important expression of HIF-1α among ischemic limb mice. It has been demonstrated that the abundance of the HIF-1α protein is decreased in ischemic tissues from aged mice and has also been linked with the downregulation of genes encoding angiogenic growth factors [61]. In this regard, Bosch-Mache et al. showed that reduced blood flow recovery among aged mice resembles the response of heterozygous HIF-1α knockout mice to ischemia [54]. Interestingly, the exogenous administration of constitutively active forms of HIF-1 into the ischemic limb was sufficient for overcoming the age-dependent impairment of ischemia-induced vascular remodeling in aged mice [54]. Thus, the ability of HIF signaling to regulate the angiogenic process may be one of the main factors in vascular aging. In this scenario, the HIF pill theory would provide a preventive treatment for vascular aging.

Some aging-related vascular diseases present a rapid course of regular age-dependent arterial changes called early vascular aging (EVA) [82]. Aging of the arterial wall vessels in humans can be quantified by measuring the pulse wave velocity along the aorta—the largest elastic artery—which represents a marker of arterial stiffness [82]. Superior techniques to evaluate EVA are the use of noninvasive procedures to determine arterial stiffness indexes, including the carotid intima–media thickness (IMT), central blood pressure, and endothelial damage parameters [82]. In this regard, EVA is also characterized by media vascular calcification (VC) in CKD. During chronic inflammation mediated by uremic toxins, VSMCs are significantly affected, becoming dysfunctional and causing VC, which may potentially be used as a biomarker for vascular age [16,83,84].

Aging is associated with transforming growth factor type β (TGF-β) inhibition via HIF-1 [85]. In addition to TGF-β signaling, there is crosstalk between the HIF pathway and well-known stress-related sensors, including AMPK (AMP-activated protein kinase), sirtuins, and nuclear factor-κB (NF-κB) [86]. Despite the role of sirtuins in the regulation of aging and longevity, nowadays, its role is still controversial [87]. Satoh et al. [87] described that sirtuin-1 activity is critical in the systemic regulation of tissue communication, aging, and longevity in mammals. Notably, overexpression of sirtuin-1 due to the calorie restriction diet plays a role in the pathogenesis of age-associated mitochondrial damage in aging kidney mice [88]. Recently, it has been demonstrated that sirtuin-1 and HIF-1α are connected [89]. In this sense, sirtuin-1 induced deacetylation of HIF-1α in aged kidneys, protecting tubulointerstitial damage [89]. Since 2012, it is well known that sirtuin-1 is necessary for HIF-1α protein accumulation [90] and the current knowledge is that sirtuin-1 could activate several transcriptional factors, such as HIF-1α, resulting in ameliorated mitochondria biogenesis and an extended lifespan [91]. In the pathophysiology of vascular aging and atherosclerosis, sirtuin-1 plays a protective role [92]. In endothelial dysfunction, the expression of sirtuin-1 is reduced promoting the manifestation of senescence in endothelial cells [93,94,95]. Moreover, sirtuin-1 modulates eNOS and NO production in vascular walls [92], playing a crucial role in maintaining vascular function and homeostasis.

Another vital player of vascular aging, which is positively regulated by HIF-1, is vascular endothelial growth factor (VEGF), a central mediator of angiogenesis. During aging, there is a defect in HIF-1 activity, yielding VEGF expression reduction and leading to the impairment of angiogenesis in response to the ischemia model [96]. Similarly, EPO is directly regulated by HIF, and lower secretions of EPO have been observed among old animals [97] and elderly patients [98]. Furthermore, the transcriptional program controlled by HIF-1 includes genes involved in many aspects of cellular homeostasis, and HIF-1 abolishment by aging could generate defects in the physiological responses to hypoxia [96]. Recently, we found that HIF-1α is involved in p53, p16, cyclin D1, and lamin B1-mediated senescence in ECs [29]. Moreover, senescent ECs failed to express HIF-1α, and the microvesicles (MVs, an EVs subtype) released by these cells were unable to carry HIF-1α [29]. In another study, HIF-1α was found to play a critical regulatory role in vascular inflammation among macrophages after intimal injury through limiting excessive vascular remodeling. The mechanism by which macrophage-derived HIF-1α mediated this effect is still unknown [99]. Considering these findings, HIF-1α may represent a possible therapeutic target in vascular diseases, especially in vascular aging.

## 5. HIF and Atherosclerosis

Although atherosclerosis has been considered chronic inflammation, intensive research in recent years has shown that it can also be considered an age-related pathology [28,100]. Many pieces of evidence have demonstrated the role of vascular senescence in atherogenesis [25,101]. We briefly mentioned above that senolytic drugs (anti-senescence) have been proposed as a therapeutic option for cellular aging and for treating human atherosclerosis [25,28]. However, although gerontologists have affirmed that atherosclerosis is associated with the characteristic features of aging in humans, cardiologists believe that aging is not a risk factor for atherosclerosis. This controversial subject was re-evaluated by Minamino *et al.*, who demonstrated that senescent vascular cells accumulate in human atheroma and that vascular cells present features of dysfunction [102,103]. These and other findings suggest that cellular senescence contributes to atherosclerosis, which is a characteristic of aging in humans.

As a model for premature aging disorder, atherosclerosis is the most common type of vascular aging where the cell vessels are susceptible to damage. Adding to the complex scenario for atherosclerosis, many studies suggest that ECs and VSMCs change and acquire features of senescent cells [104]. Moreover, during aging, blood vessels experience changes in compliance and release pro-inflammatory factors that promote atherosclerosis. Aging is associated with chronic low-grade inflammation that affects vascular and endothelial cells within the vascular wall during atherosclerosis. It is reasonable to believe that low-grade systemic inflammation may facilitate the senescent phenotype of ECs, which also contributes to the local inflammatory environment by SASP. These aging endothelium walls impair angiogenesis and decrease coagulation activity [104]. In an aged endothelium, senescent ECs failed to achieve HIF-1α stabilization and decreased miR-126 levels, which are both essential contributors to the maintenance of endothelium homeostasis [29].

In the vasculature, HIF-1α regulates pressure changes due to the negative regulation of TGF-β in ECs [85] (note that pressure overload leads to increased myocardial O_2_ consumption). In this regard, the anoxemia theory is defined as a condition of abnormal oxygenation of the arterial blood. This theory proposes that an imbalance between the demand for and supply of O_2_ in the arterial wall is a critical factor in the development of atherosclerosis [105]. As a consequence, macrophages become apoptotic, a necrotic core is built, and there is an eventual increase in angiogenesis, linking senescent cells to atherosclerosis progression [7]. Therefore, the anoxemia theory is postulated to explain the progress of atheroma plaque.

**Figure 2:**
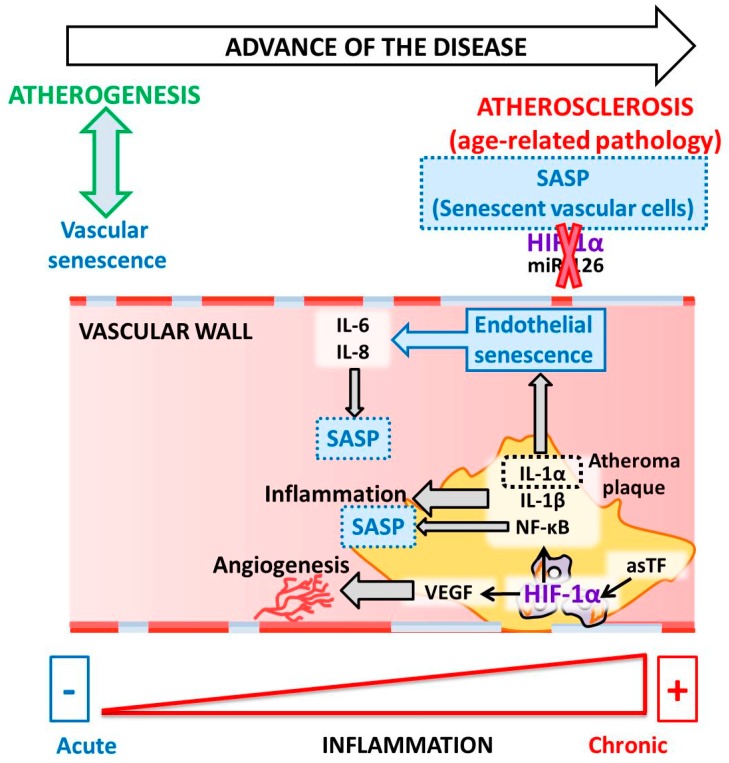
Molecular pathways linking HIF-1 to senescence during atherosclerosis.

In 2007, the presence of HIF-1α was described in atheroma plaque [106]. HIF-1α is a regulator of angiogenesis and inflammation in atherosclerotic plaque destabilization. Moreover, HIF-1α is associated with an increase in VEGF levels during the inflammatory process in atheroma plaque. Notably, activated macrophages in atherosclerosis have been observed to stabilize HIF-1α under normoxic conditions [106]. HIF-1α stabilization occurs due to the local relative hypoxia resulting from insufficient O_2_ diffusion in the thickened intima and increased O_2_ demand due to the local inflammatory response. If the O_2_ supply is restored, HIF-1α is degraded, which reduces VEGF production and subsequent angiogenic signaling [107]. Another study reported that HIF-1α increases as a consequence of neovascularization in complicated human atherosclerosis among human carotids, as well as in coronary plaques [105]. Mechanistically, the angiogenic effect of the alternatively spliced tissue factor (asTF) activates HIF-1/VEGF signaling [41]. Indeed, activated macrophages localized in atheroma plaques expressed HIF1-α and VEGF, confirming that both are critical to the regulation of human plaque angiogenesis and lesion progression. Therefore, HIF-1α mediates inflammation by promoting pro-inflammatory cytokine expression and, consequently, inflammatory cell recruitment [108,109]. In macrophages, HIF-1α regulates the expression of one of the major pro-inflammatory cytokines, IL-1β [110]. Pro-inflammatory and pro-angiogenic activities are induced in endothelial cells exposed to IL-1β stimulation [111]. Notably, anti-inflammatory IL-1β therapy led to a significantly lower rate of recurrent cardiovascular events [112]. Another IL-1 family member, IL-1α, is mainly associated with endothelial cell senescence and atherosclerosis [113]. Both IL-1α and IL-1β are minor components of SASP; however, these two cytokines are essential for boosting IL-6 and IL-8, which are secreted in large quantities by senescent endothelial cells [114,115] (Figure 2).

Moreover, extensive crosstalk between HIF and two master regulators of the inflammatory response, NF-κB and the signal transducer and activator of transcription 3 (STAT3), has been reported [108,116,117]. Under hypoxic conditions, canonical NF-κB signaling activates HIF-1α through the interactions between p50 and p65 subunits and responsive elements in the promoter of the HIF-1α gene [118]. The crosstalk between HIF-1α and NF-κB signaling during senescence has been investigated in several contexts, and its role in orchestrating SASP is widely accepted in atherosclerotic plaques. However, the full mechanism of this crosstalk is not yet completely understood.

Strikingly, Minamino et al. [104] demonstrated the presence of senescent vascular cells in human atherosclerotic lesions but not in non-atherosclerotic lesions. This study characterized some of the features of senescent cells and described an increase in pro-inflammatory mediators, including NF-κB signaling-dependent mediators, but a decrease in eNOS. In addition to these findings, signs of cellular senescence have also been detected in premature aging mouse models [119]. Together, these results provide in vivo evidence that links cellular senescence to organismal aging [104]. For instance, arterial remodeling during atherosclerosis progression is accelerated during aging. The accumulation of lipids in the arterial wall, followed by foam cell formation, is a response to endothelial damage and inflammation. Once the atheroma plaque is stable, it becomes an advanced plaque with increased susceptibility to rupture, leading to thrombosis [120].

Moreover, unstable plaque highlights the relationship between atherosclerosis and HIF-1α in ECs [121] and macrophages [122], independent of their origin (SASP or SIPS). It was also shown that the final step in atheroma plaque development is VC formation [7,123,124]. Aging causes calcification in vascular smooth muscle cells, which occurs independent of inflammation but causes arterial stiffening [120]. In this way, atherosclerosis, as well as aging or age-related atherosclerosis, causes vascular wall senescence and, as a consequence, VC—the final step of the pathology process. Increased VC in atherosclerosis produces numerous marked vascular effects, such as a reduction of tissue perfusion, which eventually causes end-organ damage, particularly in the elderly population [125].

EVs are essential modulators of vascular cell functions relevant to vascular inflammation and atherosclerosis [126,127]. Furthermore, EVs have been identified as cell-to-cell communicators. EV content includes proteins, lipids, and nucleic acids that are transferred to target cells [15,128] and modulate cell functions and phenotypes [129]. The abundance of EVs and the release of their cargos are augmented under inflammatory [129] and pathological conditions, including CVD, metabolic disorders, atherosclerosis, and DMII [129,130]. Platelets liberate EVs from vascular vessels (ECs and smooth muscle cells), erythrocytes, and leukocytes [131,132]. EVs may be potential biomarkers and pharmacological targets for atherosclerotic diseases and, therefore, may also be biomarkers for age-associated diseases, especially for EV-based therapeutics.

## 6. HIF and Endothelial Cells

One of the essential components of the vasculature are ECs. Needless to say, the dysfunction of vasculature ECs is associated with aging and chronic diseases related to aging. This age-associated endothelial dysfunction is a critical antecedent of CVD [18]. Cellular senescence in adult human ECs is accompanied by increased basal oxidative stress, which is characterized by high reactive oxygen species (ROS) production and excessive pro-inflammatory cytokine release [133]. Moreover, senescent endothelial cells release EVs that contribute to facilitating the onset of other pathologies, such as VC [7,134]. Current knowledge on endothelial aging shows that in aged sedentary subjects, ECs upregulate p53, p21, and p16^Ink4a^ protein levels, which are reduced among aged exercising adults [69], thereby linking metabolism and cellular energy consumption with the senescent phenotype of vascular ECs. These findings are consistent with the pathological role of endothelial cell senescence in age-related disorders, including obesity, diabetes, and heart failure [18,69,135].

Evidence suggests that HIF-1 increases oxygenation of hypoxic tissues and, as a consequence, promotes endothelial angiogenesis and migration through a transcriptional program that leads to increased VEGF production [136,137]. Besides its role as an essential factor in VEGF-mediated effects on ECs, HIF-1 also has critical cell-autonomous functions in ECs [43,85]. In this regard, HIF participates in the formation of new vessels, enhancing the delivery of oxygenated blood to tissues [63]. In support of this, ECs under hypoxic conditions generate tube formation, which is negatively regulated by melatonin treatment. Melatonin, a well-known natural hormone whose levels decline with age, decreases cellular and secreted VEGF levels and tube formation as a result of a reduction in HIF-1α protein expression, nuclear localization, and transcriptional activity [138]. Moreover, under hypoxia, ECs display their typical morphology and phenotype, whereas treatment with melatonin prevents this effect and cells do not present their endothelial features [138].

Further supporting the findings described above, many changes are produced during development of the senescent phenotype of the vasculature in age-related vascular diseases. In vascular pathology, senescent ECs exhibited impaired eNOS activity and decreased nitric oxide (NO) production, which are two essential mediators of the endothelial physiological function and the maintenance of vascular wall homeostasis. The alteration of these two factors is mediated by the Akt (PKB) protein. Likewise, the levels of prostacyclins, endothelial aging mediators, are significantly decreased in ECs as a consequence of age progression [104]. Altogether, endothelial dysfunction causes thrombogenesis in human atherosclerosis. Indeed, during atheroma plaque formation, the interactions between monocytes and ECs is higher when ECs have a senescent phenotype, resulting in potentiated atherogenesis, macrophage differentiation, and foam cell formation. Senescent ECs and senescent VSMCs are found in atherosclerotic plaque, indicating that these cells are involved in the progression of plaque [8].

Our own and other work have shown that HIF-1α is required to extend the replicative lifespan and is implicated in the maintenance of the protective and repair functions of ECs [29,139]. Accordingly, miR-126 is essential for signaling in endothelium homeostasis. However, HIF-1α inhibition does not affect miR-126 levels, while the inhibition of miR-126 results in diminished levels of HIF-1α protein. Thus, the miR-126/HIF-1α pathway plays a crucial role in the mechanisms of senescent ECs [29]. MVs contain miR-126, which is delivered into ECs to promote vascular endothelial repair [140]. Accordingly, MVs from senescent ECs carry lower levels of miR-126 than those from non-senescent ECs, implicating a loss of function of the endothelium [29]. Importantly, HIF-1α is seen to disappear in these MVs, confirming that MVs are regulators between cells [29]. Overall, MVs are functionally active and are involved in the mechanisms of aging regulation and, moreover, may be considered as biomarkers based on using MV composition.

## 7. HIF and Vascular Smooth Muscle Cells

VSMCs are the most abundant cell type in vasculature and are critical for maintaining homeostasis due to their functions in the vascular system. VSMCs are characterized by their capacity to contract and relax, as well as in vessel remodeling [141]. Furthermore, Lacolley et al. recently provided a new perspective for understanding the role of VSMCs in arterial stiffness and how interactions with ECs can regulate vascular aging [120]. As a consequence, Some VSMCs undergo phenotypic changes that lead to vascular degeneration during arterial aging. Moreover, some VSMCs do not achieve senescence and suffer proliferation. The increase in proliferative VSMCs is associated with an increased release of the extracellular matrix (ECM) to the wall, thereby augmenting vascular wall thickness and stiffness [141].

Aging *per se* and age-associated pathologies generate senescent VSMCs that undergo phenotype changes. These changes include quiescent contractile phenotypes (differentiated VSMCs) and synthetic phenotypes (dedifferentiated VSMCs) characterized by reduced expression of SMC-specific contractile proteins (α-SMA, SM-MHC, and calponin), as well as increased cell proliferation and production of pro-inflammatory cytokines in differentiated VSMCs [120,141]. At present, cells are understood to reduce their contractile capacity, migration, proliferation, and reduce ECM production. As a consequence, VSMCs change their phenotypes and are unable to maintain blood flow around the body because they lose their elasticity. Some specific contractile mediators are metalloproteinase, collagenase, osteopontin and, also, an increase in the production of EVs. When a vessel is damaged, a piece of machinery for repair is triggered, and the migration mechanism and proliferation of VSMCs are critical. Accordingly, this entails an increase in the abundance of growth factors (PDGF, TGF, and VEGF) and the volume of the ECM, with the ultimate purpose of reconstructing the vasculature following injury [120,141,142,143].

Furthermore, the behavior of VSMCs in the vasculature depends on the microenvironment in the vascular wall. VSMC proliferation may be beneficial throughout atherogenesis and not just in advanced lesions, whereas VSMC apoptosis, cell senescence, and VSMC-derived macrophage-like cells support inflammation. Therefore, the VSMC phenotype switches due to the complex structure of the atherosclerotic plaque [144]. In the final step, VSMCs accumulate pro-calcification factors like osteogenic gene expression and calcification-related proteins (prelamin A and lamin A) that may be critical in the mechanisms of EVA for CKD [16].

In this sense, several disorders are associated with VSMC phenotype switching, such as atherosclerosis, restenosis, aneurysm, and calcification [141,145,146,147]. Indeed, VC (defined as the accumulation of hydroxyapatite crystals in the vascular wall) generates stiffness in the vessels and, as a consequence, reduces the tubular blood flow associated with cardiovascular disease mortality [141,147]. Under normal physiological conditions, a competent defensive pathway would protect VSMCs from phenotypic differentiation and ectopic calcification [16].

VC is associated with a significant increase in all-cause mortality and atherosclerotic plaque rupture. Calcification of VSMCs in the vascular wall is the leading cause of mortality [124]. Recent work has revealed that VSMCs may exhibit three different phenotypes: calcific, adipogenic, and macrophagic [124]. The mechanisms by which VSMCs are dedifferentiated remain elusive, although it is known that media calcification is produced in atherosclerotic lesions. The significant drivers of calcification, together with atherosclerosis, are aging, uremia, and increased oxidative stress. Therefore, VCs are produced by aging and/or age-associated diseases. Durham et al. [124] have described mediators in the VC, such as specific pro- and anti-calcifying proteins, mitochondrial dysfunction, and uremic toxins. EVs are also novel and recently discovered determinants of VC [134].

In addition to the previously discussed mediators, when VSMCs release inflammatory cytokines, they induce SASP, and VSMCs became senescent cells. Senescent VSMCs increase cytokines and chemokines driven by secreted interleukin-1α (IL-1α), while matrix proteins cause endothelial damage [64]. Although VSMC proliferation is implicated in atherogenesis, senescent VSMCs appear in advanced plaques cultured from other plaques, thereby suggesting that VSMC senescence promotes both atherosclerosis and features of plaque vulnerability. In this way, the prevention of senescence has been identified as a potential target for intervention [148,149].

Accumulating evidence supports the contribution of the HIF pathway to the pathogenesis of diseases affecting the vascular wall, including atherosclerosis, arterial aneurysms, pulmonary hypertension, vascular graft failure, chronic venous disorders, and vascular malformation [44]. The HIF-1α protein is expressed in young VSMCs in vitro, while it is reduced in old VSMCs [96]. To date, HIF-1α has been involved in the proliferation, migration, and morphological changes of VSMCs [150,151]. The mechanism by which hypoxia induces HIF-1α expression in human VSMCs involves PI3K/Akt(PKB) and osteopontin, as well as suppression of the expression of some proteins, including AEG-1, α-SMA, and SM22α [150]. However, the main function of HIF-1α is the inhibition of the proliferation and migration of human VSMCs, indicating that HIF-1α induces VSMC phenotype switching [150]. However, stabilization of the HIF-1α protein in 9-month-old WT mice promoted increased endothelial permeability [152].

**Figure 3:**
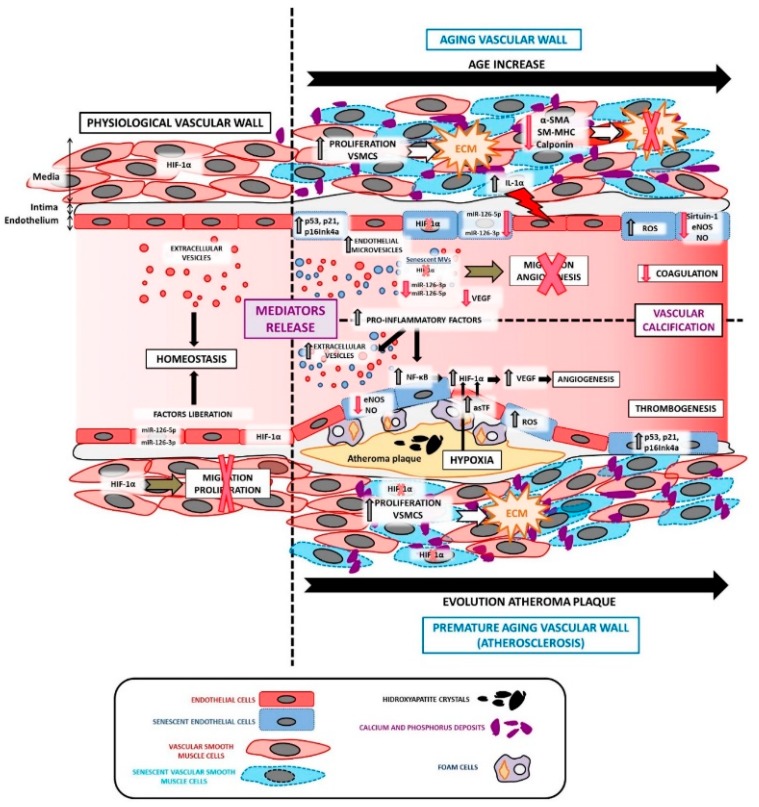
A comparison of a physiological vascular wall versus a pathological vessel as a consequence of aging *per se* or vascular age-associated diseases, as well as the reported roles of mediators in vascular aging *per se* and age-associated diseases like atherosclerosis. A brief schematic representation of the reported effects of the circulating mediators on different processes in atherosclerosis (vascular age-associated disorder) development is also presented. The mentioned effector molecules are merely examples, and it should be noted that many more exist.

As already described, Figure 3 compares a physiological vascular wall with a pathological vessel as a consequence of aging *per se* or vascular age-associated diseases, such as atherosclerosis.

## 8. Conclusions

In this review, we have provided an overview of the functional role of cellular senescence and HIF-1α implications for vascular functions in advanced age and/or aging-associated diseases, with a particular focus on atherosclerosis. Further understanding of the mechanisms underlying cellular senescence will provide new insights into the pathogenesis of age-associated vascular disorders, such as atherosclerosis. Moreover, the prevention of senescence as a potential target for intervention could be a potential anti-aging therapy. At present, senolytic drugs are being developed as a new form of therapy to extend the human lifespan. Therefore, HIF overexpression could be a new target in the strategy for senolytic drug progress. In this way, the pharmacological modulation of HIF offers a new perspective on preventing the development of endothelial cell senescence during the early phases of diseases associated with cell senescence, such as atherosclerosis, especially in vascular aging.

## 9. Perspectives

The possibility of preventing endothelial damage associated with senescence opens new perspectives in matching it with diseases, such as atherosclerosis. Currently, the primary therapeutic tool against pathologies such as atherosclerosis is based on preventing exposure to cardiovascular risk factors to prevent their harmful activity. This strategy, which so far is effective in reducing mortality associated with these diseases, seems to have reached a limit in its efficacy, as evidenced by the high morbidity that these pathologies continue to generate. One of the reasons that could explain this inertia is the fact that damaged factors, such as cellular injury associated with age, can only be partially corrected with current therapeutic approaches. Expanding the study of the mechanisms that regulate endothelial senescence should allow the development of new therapies, such as those based on the use of senolytic drugs, which allow avoiding the initial stages in the development of pathologies, such as atherosclerosis, and therefore treating patients and subjects at risk. Nevertheless, this reality is paired to deepen the knowledge of the mediators that regulate the process of cellular senescence. In this review, we present on how HIFs are essential, since these molecules are still physiological signaling elements in several cells that could be affected by new therapies; so, characterizing their physiological and/or pathophysiological activity seem to be essential to progress in the clinical use of therapies against cellular senescence.

## Figures and Tables

**Table 1:** The role of hypoxia-inducible factors in cardiovascular aging.

HIF Levels	Observations		Reference
	Pathology/Effect	Tissue/Cell Type	
↑	Ischemic cardiovascular disease	Ischemic limb (ischemic calf muscle) (young WT mice)	[54]
↓		Ischemic limb ischemic calf muscle (aging WT mice)	
↓	Vascular remodeling	Femoral artery ligation in aging mice	[53]
↓		Femoral artery ligation in *Hif1a*^+/−^ mice	
↑ (short-term)	Heart homeostasis	Heart	[52]
↑ (long-term)	Cardiomyopathy		
↓	Atherosclerosis (Premature aging disease)	Pathogenesis of plaques (Promote angiogenesis, production of factor pro-atherosclerosis and recruitment of inflammatory cells)	[3]
↓	Replicative senescence *in vitro*	Endothelial cells	[29]
